# A Review on the Prevalence of Poor Mental Health in the Construction Industry

**DOI:** 10.3390/healthcare12050570

**Published:** 2024-02-29

**Authors:** Rachel Blair Winkler, Campbell Middleton, Olivia Remes

**Affiliations:** Department of Engineering, Laing O’Rourke Centre for Construction Engineering and Technology, University of Cambridge, Cambridge CB3 0FS, UK

**Keywords:** mental health, anxiety, depression, stress, prevalence, epidemiology, vulnerable population, construction, workplace

## Abstract

A plethora of studies on the prevalence of poor mental health have been undertaken in the general population. Nevertheless, an understanding of the prevalence of poor mental health in the context of high-risk settings, such as construction, is missing. This is noteworthy as poor mental health is widespread in this context. Given that over 100 million people work in construction on a global scale, a better understanding of the burden of poor mental health in construction is needed. To this end, a review on the prevalence of key mental health-related conditions in construction was undertaken. Through this review, over 1000 papers were identified through PubMed and Google Scholar. After applying inclusion and exclusion criteria, a final set of 19 documents were included. Results showed that anxiety and depressive disorders, as well as psychological distress, have a high burden in construction. Vulnerable population subgroups (e.g., women, minority ethnic groups) were identified. Construction is a high-risk setting and despite this, the burden of poor mental health in this context is not given the importance it deserves. This review sheds light on the prevalence of key conditions, which are linked to high humanistic and economic burden. This review provides a foundation and useful starting point for further investigations, and results from this review may be used to inform future research, workplace interventions, and policy.

## 1. Introduction

According to the International Labour Organisation, there are approximately 220 million people around the world working in construction [1,2]. According to the International Trade Union Confederation, more than two million employees will be needed to work in construction in Europe by 2030 [1]. Given the upskilling and reskilling that will be taking place in construction, the need to attract new employees, and climate law targets of the industry, the mental health of workers has become more important than ever.

To show the importance of leaning into mental health in construction, estimates suggest that building and construction is the industry with the third-highest stress levels and with high rates of mental health-related conditions. In the UK, 82% of workers report stress [3,4]. Among bricklayers in the Netherlands, 18% experience depression and 11% experience posttraumatic stress disorder (PTSD) [5]. In North America, specifically in the USA, 16% of construction workers show substantial mental distress [6]. Taking this together, it appears that poor mental health is a major problem in this industry in several countries.

Studies conducted in Asia highlight similar trends. For instance, in Korea, two out of five construction field workers experience depression and trait anxiety [7]. In India, high or extreme stress is a key problem [8]. Among construction employees in China, the prevalence of medium to extremely severe levels of depression, anxiety, and stress has been reported to be higher than in other employee populations in this country [9]. A study on the construction workforce in South Africa showed that the general wellbeing of the sample was slightly above the cut-off of 50, which corresponds to poor wellbeing. The authors of the South African study concluded that future work needs to examine common mental disorders and depression symptoms specifically in construction [10]. In fact, depression is a determinant of suicide, which is a major problem in this sector.

Poor mental health needs to be taken into account because otherwise, it can increase the risk of suicide. The Branz Research Institute reports that “the construction industry has the highest proportion of suicides across all industries in New Zealand” [11]. In the UK, the suicide rate is three times higher among men working in this industry compared to the general male population. In the USA, an increased risk of death has also been reported among blue collar construction workers [12]. Despite the data on suicide rates, there is insufficient awareness regarding the extent of suicide ideation—a key determinant of the former.

When workers are having a tough time, this is not just an issue that affects those involved. It affects their families, communities, and the organisations that employ them. Poor mental health has been linked to employee disengagement and staff turnover. Mace, a global consultancy and construction firm, highlights that poor mental health costs UK employers about GBP 56 bn each year [13]. And this is important for construction companies to be aware of, as they stand to lose talent if the situation persists. In Australia, 52% of construction industry leaders agree with the following statement: “Skilled workers are leaving the industry due to the levels of stress and burnout they’re facing” [14]. Despite potentially greater public awareness related to mental health and wellbeing, the situation is not improving in construction—in fact, it could be getting worse [15]. As such, further insight is needed into key issues that are negatively affecting millions of people around the world: those working in construction.

While work has been published and publicised on stress or general wellbeing in this context, further insight into specific mental disorders is needed.

Not enough research has been conducted to synthesise information on the prevalence of mental disorders and related aspects, and we would like to address this gap. Also, there is insufficient awareness about the presence of conditions such as posttraumatic stress disorder and anxiety disorders, among other problems, in construction; we would also like to shed light on this. Employees in this industry are exposed to dangerous, transient jobs and high pressures; therefore, examining their mental health is vital. According to the Health and Safety Executive, Britain’s national regulator for workplace health and safety [16], construction is linked to the highest number of employees killed in accidents that result in death [17]. When workers are killed in fatal accidents, this can have a negative ripple effect on the mental health of the team and wider organisation. As such, to understand and bring to light the extent of poor mental health in construction, we would like to synthesise work on the prevalence of key mental health-related conditions published in the last decade.

### 1.1. Theoretical Background

#### 1.1.1. Evidence Synthesis

This review attempts to synthesise information that was published in the last ten years in key databases. While a number of studies have been conducted on the burden of stress or anxiety in construction, there are insufficient syntheses to understand the burden of a range of key conditions in construction. For example, a systematic review has recently been conducted in the context of mental health in construction; however, the focus was on risk and protective factors rather than the burden of key conditions [18]. Also, while individual studies or industry reports exist on the proportion of people affected by stress or anxiety, selective citation of these percentages does not accurately show the whole picture regarding mental health in this sector. We need to understand the burden of a range of factors spanning from stress to posttraumatic stress disorder, anxiety disorders, and others. Therefore, through our evidence synthesis, we would like to address this gap.

#### 1.1.2. Prevalence

This review examines the prevalence of key issues. Prevalence refers to the proportion of people with a certain characteristic at a point in time. Through this synthesis, we identified the prevalence of anxiety disorders, depressive disorders, posttraumatic stress disorder, and general psychological distress.

#### 1.1.3. Anxiety Disorders

This evidence synthesis captured prevalence data on anxiety disorders, which are characterised by “excessive fear and anxiety” [19]. Anxiety disorders can have a negative impact on daily functioning, and an example is posttraumatic stress disorder. This anxiety disorder occurs as a result of an event that is stressful or frightening, “or after a prolonged traumatic experience” [20]. Given that construction workers are often exposed to dangerous environments, this can be traumatic in itself [21]. Highly stressful events and trauma are interlinked and need further investigation.

#### 1.1.4. Depressive Disorders

A key aspect of depressive disorders is the loss of pleasure or interest in activities that once used to bring joy; this state of anhedonia occurs over an extended time period. Symptoms of depression can include feelings of guilt or worthlessness, thoughts of suicide, disrupted sleep, and problems concentrating [22]. Major depressive disorder has been a commonly reported and studied problem in the general population, and we would like to assess its prevalence in construction.

#### 1.1.5. Psychological Distress

According to the American Psychological Association Dictionary of Psychology, psychological distress is “a set of painful mental and physical symptoms that are associated with normal fluctuations of mood in most people” [23]. This is another factor that we included in our review.

#### 1.1.6. Rationale for Conducting This Evidence Synthesis

This evidence synthesis was undertaken to pull together work on the prevalence of key mental health-related conditions in the field of construction. Mental health is a key issue in this field and there is insufficient research comprehensively synthesising work on this topic.

## 2. Materials and Methods

On 11 May 2023, a Wellbeing in Construction forum took place at the Laing O’Rourke Centre for Construction Engineering and Technology at the University of Cambridge, focusing on the mental health of construction workers. It emerged that poor mental health (e.g., high anxiety levels) and related issues, such as stress, are key problems among those working in construction and need further examination. Attendees included employees from major construction companies in the UK and academics from various parts of the world. This helped to inform the need for conducting this review of the evidence.

The Wellbeing in Construction forum was complemented by a review of various industry reports and academic articles to identify the topics for focus of this synthesis. The Lancet, one of the most cited medical journals in the world [24], suggests that work-related stress, anxiety, and depression have a major negative impact on the construction industry. In fact, stress, anxiety, and depression are linked to a yearly cost of about 400,000 work-days in the context of UK construction [25]. This suggests that these factors are worth examining further through a synthesis of the evidence. Determining the number of people affected around the world helps direct the targeted allocation of scarce health resources, among other things.

Stress, depression, and anxiety have also been highlighted as issues by a major construction industry charity [26]. Various consultancies, such as Refocus Safety Ltd., which focuses on construction health and safety, further suggest that the construction environment can be fertile ground for traumatic experiences, and posttraumatic stress disorder, in particular, may be present among workers [27]. Taking all this together, we focused our review on these key aspects. To provide a more complete picture of mental health in construction, we expanded our searches beyond stress, anxiety, and depression to also include suicide ideation and psychological distress. Suicide ideation can be an important warning signal for suicide, an issue in construction around the world. Furthermore, the high job demands in construction can take a psychological toll and lead to high levels of distress among the workers; this aspect also emerged during the Wellbeing in Construction forum. A study by Dr. Jack Dennerlein, an affiliate of the Center for Work, Health, and Well-being at the Harvard T.H. Chan School of Public Health, shows that about a third of construction workers in the USA experience mental distress [28]. Given the commonality of such issues in various countries around the world, further work is needed to understand the overall burden of key mental health-related conditions in construction. In order to understand the full extent of the problem and avoid generalisations based on highly select studies, a global synthesis is needed.

### Inclusion and Exclusion Criteria; Search Strategy

A review of academic evidence from the past 10 years was undertaken. In several aspects, we attempted to conduct this review according to PRISMA extension for scoping review (PRISMA Sc-R) guidelines [29]. The inclusion and exclusion criteria are described below.

- Inclusion criteria:
  − We allowed for the inclusion of a range of documents in our synthesis, such as primary studies, reviews, etc. We were interested in documents capturing prevalence, and the population of interest was construction employees. The umbrella term of “poor mental health” that we use throughout this document captures outcomes that are of relevance to public health and factors that have been reported to be of key concern in construction. This includes anxiety disorders, depressive disorders, psychological distress, trauma, and suicide ideation (a major risk factor of suicide). This allowed us to keep the review focused, but at the same time provide a high breadth and depth of information;
  − We included documents written in the English language;
  − We captured research on the prevalence of poor mental health in construction.
  − We searched for literature conducted across the globe and with participants employed in a range of roles in construction.
- Exclusion criteria:
  − We excluded qualitative literature;
  − Documents that focused on substance abuse were excluded, as this extensive research area would be worthy of a separate review;
  − We also excluded suicide incidence, as this is a separate topic (and the aim of this review was specifically related to prevalence).

1. Following on from this, we searched PubMed (years: 2013–2023) using combinations of keywords related to construction, prevalence, as well as anxiety, depression, psychological distress, trauma, stress, and suicide ideation. To cross-check PubMed searches, Google Scholar was searched using the same keywords and date range (2013–2023);
2. We applied the previously described inclusion and exclusion criteria while sifting through the titles and abstracts of documents. One author performed title and abstract screening of the documents to assess whether they met the inclusion criteria, with a sample of articles checked by a second author;
3. Then, the full texts of the abstracts that fulfilled the inclusion criteria were obtained. Subsequently, inclusion and exclusion criteria were applied to these documents. We searched for literature that reported on anxiety disorders (single disorders or aggregated), depressive disorders (single or aggregated), psychological distress, suicide ideation, trauma, and stress. We were interested in the prevalence of these disorders or related factors, but specific to the construction industry (e.g., a study could include multiple industries but had to quantify construction-specific data). Documents were included regardless of the measuring tools used to assess prevalence. One author performed title and abstract screening of the documents to assess whether they met the inclusion criteria, with a sample of articles checked by a second author;
4. Next, data extraction took place.

Data extraction was performed, and a standardised data extraction form was created to capture the following: author(s), publication date, geographic region of the study, population demographics, sample size, method of determining prevalence, results specific to prevalence, as well as limitations and directions for future research as reported by the authors of the studies/documents. The bibliographies of papers were also hand-searched. A dedicated Google Drive and Google Sheets were used to store data and record every phase of the data search, collection, and extraction. A subsample of papers was cross-checked by the second author.

## 3. Results

**Figure 1:**
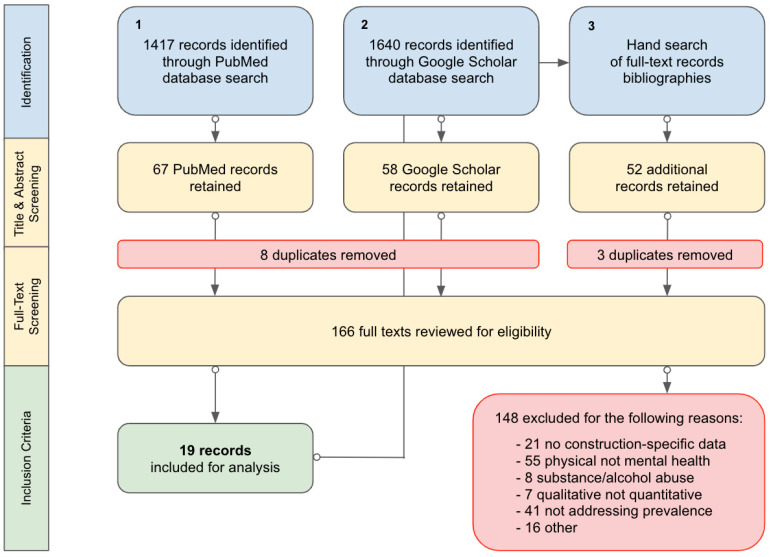
Flowchart of the review process.

We identified over 1400 records through PubMed and over 1600 records through Google Scholar; hand searches revealed 52 additional records (Figure 1). Following record identification, document retention and deduplication, and hand searches, we were left with 166 documents. We reviewed the full texts of these documents according to the inclusion and exclusion criteria, and following this phase, identified a final list of 19 papers. These were primary studies and included populations originating from Nepal, the Netherlands, South Africa, Australia, the USA, China, South Korea, Singapore, Malaysia, Bangladesh, India, Myanmar, Thailand, and the Philippines. Table S1 includes the data extracted from the 19 documents.

### 3.1. Description of Research Included

Of the 19 studies identified, 11 included data on depression, 10 on mental stress or psychological distress, 4 on anxiety, 4 on suicide, and 1 on posttraumatic stress disorder (PTSD). The documents captured assess psychopathology or related factors using 14 different methods of measurement.

### 3.2. Psychological Distress

A number of these studies measured psychological distress using tools such as the Kessler 6 and Kessler 10. Of the studies included in this review, the prevalence of psychological distress in construction ranged from 5–7% in construction populations (5% for bricklayers and 7% for supervisors) in the Netherlands [5] to 60% in Australia [30] (60% corresponds to those who reported ‘moderate’, ‘high’, or ‘very high’ psychological distress levels on the K10; the sample included remote construction workers). These findings suggest that a substantial proportion of construction workers are at risk of poor mental health.

### 3.3. Depression and Anxiety

This review identified one study that included data on PTSD. The study, conducted in the Netherlands, used the Dutch employee register to identify a sample of construction supervisors and field workers (1500 participants were selected). The study found that 11% of the field workers and 7% of construction supervisors experienced symptoms consistent with PTSD [5]. The study suggested that PTSD contributes to poor mental health in construction.

Depression was the most frequently measured mental health condition in this review, and the prevalence of this condition was assessed using different methods. The adjusted prevalence of depression ranged from 8% to 12% in research that measured the latter condition via ICD-9-CM diagnosis codes [31,32]. Also, a comprehensive study using 18 years of data from the US Bureau of Labor Statistics measured a prevalence of depression in construction worker populations; the point estimates corresponding to CES-D scores of 10–21 ranged between 6% and 12% [31]. A regional US study compared measurements from different verticals of construction and found the lowest prevalence (8%) in heavy civil contractors and the highest (12%) in specialty trade contractors [31].

Studies using self-report screening tools tended to show a higher prevalence of psychopathology in the context of construction. At the lowest end of the range, a regional study of 402 Nepalese construction workers measured a 17% prevalence of depressive symptoms [33]. At the highest end, a national study of 430 Korean field workers measured a 38% prevalence of depressive symptoms [7]. Four other studies identified a high burden of depression in various settings worldwide. One study measured 17.6% and 19.6% in bricklayers and construction supervisors, respectively, in the Netherlands [5]. Another showed a 29% prevalence of depression in Singapore construction companies that employed workers from Bangladesh, China, India, Myanmar, Thailand, Malaysia, and the Philippines [34]. A third study highlighted a prevalence of 33% (mild-moderate and severe depression) in Australian construction industry professionals [35] and a fourth estimated a prevalence of 38% in Korea [7]. In addition, depression prevalence estimates of 30% and 37% were found by two nationally representative samples in China [9,36].

In addition to depression, the documents included in this review also found a high prevalence of anxiety symptoms in construction populations in different parts of the world. A regional Nepalese study of workers on building construction sites measured a 19.2% prevalence of anxiety [33]. In construction participants living in China, moderate to extremely severe anxiety was measured at a prevalence of 33.6% [9]; in Australia, mild/moderate to severe anxiety was measured at a prevalence of 36% [35]. At the highest end, Lim et al. (2017) [7] estimated that 42.7% of construction workers in Korea experienced symptoms of trait anxiety; the study investigated those working on road, bridge, tunnel, subway, and apartment construction sites. In sum, the evidence base on anxiety shows that the prevalence of this condition is high among construction employees in regions around the world.

### 3.4. Suicide Ideation

An increased risk of suicide in the construction industry has been identified in many countries.Most studies quantifying the impact of suicide focus on incidence rate, not prevalence, and are, therefore, excluded from this review. There are, however, studies which measure the prevalence of suicidal ideation, an important risk factor. Tyler et al. (2022) [37] found that 7.3% of Australian men in construction have suicidal ideation, and more concerningly, Ross et al. (2022) [38] measured a 29% prevalence in Australian apprentices (the majority of apprentices being aged 18 to 25 years). These studies illuminate the burden of suicide ideation in construction.

### 3.5. Vulnerable Subgroups

Other findings highlight that subgroups of construction worker populations, namely women, ethnic minorities, and those of migrant status, may be at an increased risk of poor mental health. Kamerdeen and Sundindiji’s (2017) analysis revealed that the prevalence of anxiety and depression in men was similar to normative Australian data; however, there was comparably higher prevalence and severity of both anxiety and depression symptoms in females, especially those in management positions [39]. In Asia, migrants and ethnic minorities tend to have a measurably higher prevalence of severe psychological distress than the rest of the populations studied in construction. Taken together, these results suggest that population subgroups in construction can have an increased risk of poor mental health.

### 3.6. Summary of Limitations and Recommendations

This section highlights the limitations and recommendations as reported by the authors of the documents included in this paper.

In this review, 15 of 19 studies self-identify cross-sectional research design as limiting to understanding causation and the impact of contributing and mitigating factors over time. Also, seven of the nineteen studies were published after 2020. Some studies identify the potential impact of COVID-19 on psychological wellbeing, which could have contributed to poor mental health unrelated to construction. Both limitations support the need for longitudinal studies where changes in mental health can be measured over time. This would also allow for prospective research to test the effectiveness of specific intervention strategies.

Another limitation is that many of these studies are quite broad across the verticals and trades of construction. While this allows for larger sample sizes, it limits our ability to understand specific variables (e.g., job roles and associated risk factors) contributing to poor mental health. For example, day-to-day physical hazards, and therefore mental stressors, may be different for an electrician versus a mechanical contractor. Cultural influences specific to certain migrant populations may also impact mental health outcomes differently. Therefore, more granular research by job and demographic profile could inform intervention programmes, which this research suggests the industry is in need of. Finally, the studies call for rich qualitative data to understand the contributing and mitigating factors relevant to individual wellbeing in construction.

## 4. Discussion

This review identifies a 7–11% prevalence of PTSD, a 10–38% prevalence of depression, a 19–42% prevalence of anxiety, and a 21–60% prevalence of moderate to severe psychological distress in construction workers around the world.

### 4.1. Theoretical Contributions

The synthesis of evidence published in the last ten years shows that the burden of poor mental health is high in construction workers around the world. Estimates from employees originating from countries such as the Netherlands, Korea, the USA, Nepal, Australia, Singapore, and other parts of the world show that depression is particularly problematic in this sector. In fact, one of the highest prevalence estimates of depressive symptoms was reported to be 37.6% in Korea. The reason for the high burden of depression relates to the difficult working environment of construction workers: the authors of the Korean study highlight the “psychological and physical” demands in this sector [7].

Anxiety was found to be high in Australia and it was an issue among female professionals at middle management levels [39]. Construction can be stressful, and it may be particularly problematic if social support networks are missing. Construction in Australia (and other parts of the world) is male-dominated and lacks gender diversity, which can have an impact on workers’ mental health.

This synthesis shows that the burden of stress was found to be high in construction workers in Singapore; moderate to extremely severe levels of stress were reported for 33% of participants. In Singapore, a number of construction employees originate from other countries. According to the researchers, it can be challenging to be a worker of migrant status [34]. Among migrant workers from Bangladesh employed in construction in Singapore, psychological distress is an issue and has been linked to healthcare access barriers [34]. Working in another country often comes with its own set of challenges. On top of this, when adverse working conditions are added to daily life, this can be stressful and linked to psychological distress.

### 4.2. Practical Contributions

Research recognises the need for treatment of depression among construction workers. Psychological specialists are needed to diagnose and provide plans of action for those who are struggling [7]. Researchers from the University of New South Wales Sydney discuss the need for creating workplace programmes that provide psychological support and facilitate communication between employees [35]. Small things, such as “corridor conversations” or “coffee break chats”, can also go a long way in supporting people’s mental health [35]. It also appears that social support coming from management is particularly beneficial [28,40].

While focusing on health promotion strategies is key, it is equally important to address the harmful determinants of mental health. It is vital to address factors such as discrimination and harassment in the workplace. Discrimination, for example, has been linked to psychological distress in the context of construction [28]. As such, resources and plans of action to tackle harmful determinants in the workplace are necessary. Having a mental health plan at work, resources dedicated to carrying out this work, and awareness days to improve knowledge about the importance of mental health among construction staff are important aspects for the industry to consider.

In order to improve work–life balance and mental wellbeing, organisations within the industry need to allow for greater flexibility and have better communication among staff at different levels. For example, managers should allow for greater flexibility in taking days off to care for sick family members, and efforts should be made to minimise unpredictability around work schedules (e.g., clearer communication from the top down).

### 4.3. Comparison of Construction with Other Industries

We can learn from other industries which have struggled with poor mental health among their employees. The healthcare industry is one such example, and the long working hours and high work demands are key contributing factors to negative outcomes [41]. In fact, the British Medical Association highlighted this year that stress levels among doctors are very high [42]. The dental industry is a sector known for its challenges. A recent systematic review focused on interventions [42], highlighted the anxiety, stress, and burnout that dentists experience. Despite this, there is insufficient work on mental health interventions for dentists or the effectiveness of such initiatives; this mirrors the mental health landscape in construction [42].

The Office for National Statistics (ONS) has shown that the risk of suicide is particularly high among low-skilled male labourers, and construction is highlighted by the ONS as an industry giving rise to this. Plasterers, painters, and decorators are groups that have an increased likelihood of such negative outcomes [43]. Common mental health problems, such as anxiety and depression, as well as stress, can factor into this. And poor mental health can also lead to days off sick. However, other characteristics of challenging occupations can lead people on a downward spiral. For example, cleaning personnel may be faced with job insecurity, problems with pay, and unpredictable work schedules, which are also characteristics of construction [43]. It appears that occupations and industries with high levels of poor mental health often share commonalities. And if we begin to understand the drivers behind the high burden of anxiety, stress, and depression, we are in a better position to do something about it.

### 4.4. Strengths of Our Work

This review has a number of strengths. Its area of focus is greater than that of extant syntheses, and extensive searches have allowed us to capture settings and populations across the globe. Other work on poor mental health in construction has focused on specific at-risk populations, such as migrant workers, remote workers, and specific gender groups. Our review takes into account a range of population subgroups across the globe. Extant systematic reviews have also focused on stress to understand industry-specific risk factors for poor mental health [18,44]. This, however, still leaves a gap with respect to common mental health conditions. As this review has shown, depression and anxiety are some of the most common and burdensome disorders in the workplace, and also need to be taken into account (in addition to stress). As such, a strength of this review is the focus on the prevalence of a number of key mental health-related conditions in construction.

### 4.5. Limitations of Our Work

Besides strengths, our work also has weaknesses. One weakness is the fact that this information is not generalisable to countries and populations not included in this review. Another limitation is the use of prevalence as an inclusion criterion. Thirty-nine studies on wellbeing in construction were not included because of this. The excluded studies contain data that could add to the overall understanding of poor mental health in construction; however, this is a limitation of all reviews. While we may have missed potentially useful information because of this, the benefit of focusing on prevalence is that it allows for a narrow focus to quantify the scope of the industry problem on a global level. To combat this limitation, we conducted extensive searches and an in-depth synthesis, which allowed us to gain detailed insight into the burden of psychopathology in construction work settings.

Given the high heterogeneity in the information reported within the included documents, it was not possible to conduct a meta-analysis. Another limitation relates to the differences between the studies that were available. As a result of the heterogeneity related to the types of documents included, a systematic quality assessment of the evidence was not possible. Our aim, however, was to bring together the heterogeneous, fragmented evidence base on the burden of poor mental health in construction; as such, findings from this review can serve as an important starting point and foundation for future research.

### 4.6. Future Research

#### 4.6.1. Academia

This review highlights the need for additional research in specific areas. Researchers have noted the risk of PTSD is higher among construction workers due to hazardous working conditions [45,46], yet this review identified only one study on the prevalence of PTSD in this sector in line with our aims. Compared to other mental health conditions, PTSD is less well researched. Furthermore, previous evidence suggests an elevated risk of this condition among employees in construction. As such, further work on the burden of PTSD in the workplace is an important gap to fill.

An area which deserves a separate in-depth review revolves around substance abuse in construction. While reviewing the prevalence of substance abuse was beyond the scope of this focused review, it is a major issue that also needs to be assessed in this sector. Substance abuse, specifically excessive alcohol consumption, has been correlated to construction worker suicide [47], and is linked to depression and anxiety. A survey of Australian construction trade apprentices suggested that this population sub-group had a high risk of alcohol and drug-related harm [48]. Another survey of over 500 Australian male construction workers showed that 1 in 6 participants saw workmates “being visibly affected by alcohol in the workplace” [49]. In Korea, 38.4% of construction workers have been identified to have problems with drinking, and over 20% of the exhibited drinking behaviours classified as being alcohol abuse or dependency [7]. Other studies in various parts of the world similarly suggest that substance abuse is a key problem in construction, and further work on this can elucidate the way forward with respect to research, clinical practice, and industry wellbeing programme development.

While this review has comprehensively synthesised and provided an in-depth look at key issues in construction, further work is needed to inform programmes and interventions. Increased academic research on this topic can lead to better outcomes. Australia is an illustrative example. There is a significant amount of research that has been conducted on construction workers’ mental health in this region. In our review, six of the nineteen studies took place in Australia, where non-profit organisations focusing on mental health, such as MATES, have emerged over the last decade. Maheen et al. (2022) [50] identified a decline (3% decline in construction workers vs 1.5% in all other workers in suicide trends in the last five years in Australia, suggesting that the interventions have had a positive impact in the construction sector. In order to combat the global problem of poor mental health in construction, it is important to carry out prevalence studies in parts of the world in which such research is scarce or insufficient. For example, prevalence studies need to be rolled out in less-developed countries as well, and this could be a direction for future research.

Various extant systematic reviews assess psychopathology in construction, but with a specific aim. For example, Hutton et al. (2022) [51] evaluate the quality of methods used in existing research on psychopathology to inform clinicians on methodology for future research. Gómez-Salgado et al. (2023) [52] review the prevalence of anxiety (as well as stress and fear) in construction with the vision of improving workplace conditions. The authors of this paper argue that understanding the prevalence of poor mental health in construction is a worthy aim in and of itself. One of the key reasons is that it can inform workplace mental health and wellbeing interventions, and ultimately have a positive impact on quality of life.

#### 4.6.2. Industry

There are a few recommendations for the industry. First, in light of the high burden of poor mental health in construction, it is important to assess the mental health and wellbeing interventions that have been developed more generally for the public and determine whether they may apply in the context of construction. Second, it is necessary to determine the extent to which construction organisations use mental health promotion programmes or undertake wellbeing initiatives. Third, it is important to assess whether the strategies used to address mental health are effective. We need to find out whether strategies such as the use of mental health champions, awareness programmes, mental health first aiders, and emergency hotlines indeed contribute to a decline in levels of high stress, anxiety, and depression in construction employees. Further research is needed on this. Insight into the impact of interventions using robust measurement methods is needed.

A number of mental health and wellbeing interventions and strategies have been developed in the wider context of population health (for example, to boost hardiness, resilience, and facilitate posttraumatic growth). It would be of use to trial some of these in the context of construction and measure their influence on key outcomes.

## 5. Conclusions

In order to generate effective strategies to address poor mental health in construction, it is, first of all, important to understand the scope of the problem. Findings from this review suggest a high prevalence of poor mental health in construction and shed light on the most vulnerable populations. Multiple stakeholders can benefit from this area of research. Understanding the prevalence of poor mental health in construction can inform private sector employer assistance programmes, government policy, and industry advocate group actions. Additionally, this study helps triangulate the most critical needs for future academic and industry work. This review helps quantify the breadth of the industry-wide problem, implying a moral imperative for action in the academic community, public sectors, and private industry.

## Data Availability

Data sharing is not applicable.

## Supplementary Materials

The following supporting information can be downloaded at: https://www.mdpi.com/article/10.3390/healthcare12050570/s1, Table S1: Data extraction capturing the burden of poor mental health in construction [53,54,55,56,57].

## References

[1] International Labour Organization (2019). Developing the Construction Industry for Employment-Intensive Infrastructure Investments. https://www.ilo.org/wcmsp5/groups/public/---ed_emp/---emp_policy/---invest/documents/publication/wcms_734235.pdf.

[2] International Trade Union Confederation (2023). More Than Two Million Workers Will Be Needed in the Construction Sector in Europe by 2030. https://www.ituc-csi.org/more-than-two-million-workers-will-be-needed-in-the-construction-sector-in-europe-by-2030.

[3] AXA (2019). AXA Stress Index 2018. https://www.axa.co.uk/contentassets/d818306b7ffc4aa9beea44078ba99eb3/axa-stress-index-2018.pdf/.

[4] Gerrard N. Construction Is Third Most Stressful Industry. Chartered Institute of Building (CIOB) Construction Management. 19 November 2018. https://constructionmanagement.co.uk/construction-third-most-stressful-industry/.

[5] Boschman J.S., van der Molen H.F., Sluiter J.K., Frings-Dresen M.H.W. (2013). Psychosocial work environment and mental health among construction workers. Appl. Ergon..

[6] Jacobsen H.B., Caban-Martinez A., Onyebeke L.C., Sorensen G., Dennerlein J.T., Reme S.E. (2013). Construction Workers Struggle with a High Prevalence of Mental Distress, and This Is Associated with Their Pain and Injuries. J. Occup. Environ. Med..

[7] Lim S., Chi S., Lee J.D., Lee H.J., Choi H. (2017). Analyzing psychological conditions of field-workers in the construction industry. Int. J. Occup. Environ. Health.

[8] Bhatt R.R., Desai G.J., Verma P.B. (2015). Psychological stress among un-organized building construction workers in Gandhinagar, Gujarat, India. J. Med. Res..

[9] Zhang S., Sunindijo R.Y., Frimpong S., Su Z. (2023). Work stressors, coping strategies, and poor mental health in the Chinese construction industry. Saf. Sci..

[10] Raliile M., Haupt T., Kajimo-Shakantu K. Assessing the Mental Well-being of the Construction Workforce in South Africa using the World Health Organisation (WHO-5) Wellness Index Measure. Proceedings of the 16th Built Environment Conference.

[11] Bryson K., Duncan A. (2018). Mental Health in the Construction Industry Scoping Study (Study Report SR411). https://mates.org.au/media/documents/SR411_Mental_health_in_the_construction_industry.pdf.

[12] Hawkins D., Davis L., Punnett L., Kriebel D. (2020). Disparities in the Deaths of Despair by Occupation, Massachusetts, 2000 to 2015. J. Occup. Environ. Med..

[13] MACE Construction Industry Can Pave the Way to Better Support on Mental Health. 30 November 2022. https://www.macegroup.com/perspectives/articles/2022/november/construction-industry-can-pave-the-way-to-better-support-on-mental-health.

[14] PROCORE (2022). Press Release. Australian Construction Exodus Driven by Stress and Burnout, According to Industry Leaders. https://www.procore.com/press/australian-construction-exodus-driven-by-stress-and-burnout-according-to-industry-leaders.

[15] Garbett J., Demolishing the Stigma around Mental Health in Construction PBC Today, 8 February 2023. https://www.pbctoday.co.uk/news/health-safety-news/demolishing-the-stigma-around-mental-health-in-construction/120905/#:~:text=While%20mental%20health%20in%20construction,the%20importance%20of%20this%20subject.

[16] UK Government (2023). Health and Safety Executive. https://www.gov.uk/government/organisations/health-and-safety-executive.

[17] Health and Safety Executive (2023). Work-Related Fatal Injuries in Great Britain.

[18] Chan A., Nwaogu J., Naslund J. (2020). Mental Ill-Health Risk Factors in the Construction Industry: Systematic Review. J. Constr. Eng. Manag..

[19] American Psychiatric Association What Are Anxiety Disorders?. June 2023..

[20] National Health Service (NHS) Overview-Post-Traumatic Stress Disorder. 13 May 2022. https://www.nhs.uk/mental-health/conditions/post-traumatic-stress-disorder-ptsd/overview/.

[21] LetsBuild Why Is Working in Construction So Dangerous? 18 October 2023. https://www.letsbuild.com/blog/working-construction-dangerous#:~:text=It’s%20widely%20accepted%20that%20construction,lightning%20when%20working%20at%20height.

[22] World Health Organization (WHO) Depressive Disorder (Depression). 31 March 2023. https://www.who.int/news-room/fact-sheets/detail/depression.

[23] American Psychological Association APA Dictionary of Psychology: Definition of “Psychological Distress”. 19 April 2018. https://dictionary.apa.org/psychological-distress.

[24] Casino G., Rius R., Cobo E. (2017). National citation patterns of NEJM, the lancet, JAMA and the BMJ in the lay press: A Quantitative content analysis. BMJ Open.

[25] Burki T. (2018). Mental health in the construction industry. Lancet Psychiatry.

[26] Lighthouse Construction Industry Charity HSE (UK Health Safety Executive) Working Minds Campaign One Year On. 15 November 2022. https://www.lighthouseclub.org/hse-working-minds-campaign-one-year-on/.

[27] Refocus Safety Ltd. Understanding the Psychological Effects of Construction Work on Worker Well-Being. Construction Health and Safety Consultancy. 20 February 2023. https://refocussafety.co.uk/understanding-psychological-effects-construction-worker-well-being.

[28] Dennerlein J.T., Eyllon M., Garverich S., Weinstein D., Manjourides J., Vallas S.P., Lincoln A.K. (2021). Associations between work-related factors and psychological distress among construction workers. J. Occup. Environ. Med..

[29] Tricco A.C., Lillie E., Zarin W., O’Brien K.K., Colquhoun H., Levac D., Moher D., Peters M.D.J., Horsley T., Weeks L. (2018). PRISMA extension for scoping reviews (PRISMA-ScR): Checklist and explanation. Ann. Intern. Med..

[30] Bowers J., Lo J., Miller P., Mawren D., Jones B. (2018). Psychological distress in remote mining and construction workers in Australia. Med. J. Aust..

[31] Wulsin L., Alterman T., Timothy Bushnell P., Li J., Shen R. (2014). Prevalence rates for depression by industry: A claims database analysis. Soc. Psychiatry Psychiatr. Epidemiol..

[32] Dong X.S., Wang X., Largay J.A., Sokas R. (2015). Long-term health outcomes of work-related injuries among construction workers-findings from the National Longitudinal Survey of Youth. Am. J. Ind. Med..

[33] Adhikari B., Poudel L., Bhandari N., Adhikari N., Shrestha B., Poudel B., Bishwokarma A., Kuikel B.S., Timalsena D., Paneru B. (2023). Prevalence and factors associated with depression, anxiety and stress symptoms among construction workers in Nepal. PLoS ONE.

[34] Palaniappan K., Natarajan R., Dasgupta C. (2023). Prevalence and risk factors for depression, anxiety and stress among foreign construction workers in Singapore—A cross-sectional study. Int. J. Constr. Manag..

[35] Kamardeen I., Sunindijo R.Y. (2017). Personal Characteristics Moderate Work Stress in Construction Professionals. Pers. Charact. Moderate Work. Stress Constr..

[36] Huang L., Sun X., Zhou M. (2020). Depressive symptoms in Chinese laborers: Prevalence and correlated factors among subgroups. J. Affect. Disord..

[37] Tyler S., Gunn K., Esterman A., Clifford B., Procter N. (2022). Suicidal Ideation in the Australian Construction Industry: Prevalence and the Associations of Psychosocial Job Adversity and Adherence to Traditional Masculine Norms. Int. J. Environ. Res. Public Health.

[38] Ross D.V., Mathieu D.S., Wardhani M.R., Gullestrup M.J., Kõlves D.K. (2022). Suicidal ideation and related factors in construction industry apprentices. J. Affect. Disord..

[39] Yosia Sunindijo R., Kamardeen I. (2017). Work Stress Is a Threat to Gender Diversity in the Construction Industry. J. Constr. Eng. Manag. Asce.

[40] Petrie K., Gayed A., Bryan B.T., Deady M., Madan I., Savic A., Wooldridge Z., Counson I., Calvo R.A., Glozier N. (2018). The importance of manager support for the mental health and well-being of ambulance personnel. PLoS ONE.

[41] Burton J., Stress in the Workplace: Most Stressful Industries MQ Mental Health Research. 20 April 2023. https://www.mqmentalhealth.org/stress-in-the-workplace-most-stressful-industries/.

[42] Plessas A., Paisi M., Bryce M., Burns L., O’Brien T., Hanoch Y., Witton R. (2022). Mental health and wellbeing interventions in the dental sector: A systematic review. Evidence-Based Dentistry.

[43] Windsor-Shellard B., Gunnell D. (2019). Occupation-specific suicide risk in England: 2011–2015. Br. J. Psychiatry.

[44] Frimpong S., Sunindijo R.Y., Wang C.C. (2022). Mental Health Conditions among Young Construction Workers: A Systematic Narrative Review. Environ. Sci. Proc..

[45] Hu B.S., Liang Y.X., Hu X.Y., Long Y.F., Ge L.N. (2000). Posttraumatic Stress Disorder in Co-workers following Exposure to a Fatal Construction Accident in China. Int. J. Occup. Environ. Health.

[46] Stocks S.J., McNamee R., Carder M., Agius R.M. (2010). The incidence of medically reported work-related ill health in the UK construction industry. Occup. Environ. Med..

[47] Heller T.S., Hawgood J.L., De Leo D. (2007). Correlates of Suicide in Building Industry Workers. Arch. Suicide Res..

[48] Pidd K., Duraisingam V., Roche A., Trifonoff A. (2017). Young construction workers: Substance use, mental health, and workplace psychosocial factors. Adv. Dual Diagn..

[49] Roche A.M., Chapman J., Duraisingam V., Phillips B., Finnane J., Pidd K. (2020). Construction workers’ alcohol use, knowledge, perceptions of risk and workplace norms. Drug Alcohol Rev..

[50] Maheen H., Taouk Y., LaMontagne A.D., Spittal M., King T. (2022). Suicide trends among Australian construction workers during years 2001–2019. Sci. Rep..

[51] Hutton E.A., Skues J.L., Sullivan J.A., Wise L.Z. (2022). Mental health research in the global construction industry: A scoping review using a dual-continuum model of mental health. Mental Health and Prevention.

[52] Gómez-Salgado C., Camacho-Vega J.C., Gómez-Salgado J., García-Iglesias J.J., Fagundo-Rivera J., Allande-Cussó R., Martín-Pereira J., Ruiz-Frutos C. (2023). Stress, fear, and anxiety among construction workers: A systematic review. Frontiers in Public Health.

[53] Bowen P., Edwards P., Lingard H., Cattell K. (2014). Workplace Stress, Stress Effects, and Coping Mechanisms in the Construction Industry. J. Constr. Eng. Manag..

[54] Chapman J., Roche A.M., Duraisingam V., Ledner B., Finnane J., Pidd K. (2020). Exploring the relationship between psychological distress and likelihood of help seeking in construction workers: The role of talking to workmates and knowing how to get help. Work.

[55] Dong X.S., Brooks R.D., Brown S., Harris W. (2022). Psychological distress and suicidal ideation among male construction workers in the United States. Am. J. Ind. Med..

[56] Sellenger M., Oosthuizen J. (2017). Quantitative Analysis of Mental Wellbeing of fly-in fly-out Construction Project Support Service Workers. J. Prev. Med. Healthc..

[57] Wang C., Mohd-Rahim F.A., Chan Y.Y., Abdul-Rahman H. (2017). Fuzzy Mapping on Psychological Disorders in Construction Management. J. Constr. Eng. Manag..

