# Supplementary material for: A Review on the Prevalence of Poor Mental Health in the Construction Industry
Source: Healthcare (Basel). 2024 Feb 29;12(5):570. doi: 10.3390/healthcare12050570 (PMC10930880; doi:10.3390/healthcare12050570)
Supplement: Supplementary file 1 [file healthcare-12-00570-s001.zip › healthcare-2723250-supplementary.pdf]

Table S1: Data extraction capturing the burden of poor mental health in construction

Author	Country	Population	Sample (n)	Measurement method	Mental disorder and/or related	Prevalence
Adhikari et al. (2023) [33]	Nepal	General construction	n = 402	Depression, Anxiety and Stress Scales, DASS-21 Nepali version	Anxiety symptoms<br>Depression symptoms<br>Stress	19.2 % anxiety symptoms<br>17.1% depression symptoms<br>16.4% high stress
Boschman et al. (2013)[5]	Netherlands	1. Trade workers (Brick layers)<br>2. Construction professionals (Supervisors)	1. n = 262<br>2. n = 310	Whooley Depression Screen Impact Scale Impact of Event Scale, IES	Depression symptoms<br>Psychological distress<br>PTSD symptoms	1. Trade workers (Brick Layers)<br>- 17.6% depression symptoms<br>- 4.7% psychological distress<br>- 10.9% PTSD symptoms<br>2. Construction professionals (Supervisors)<br>- 19.6% depression symptoms<br>- 6.8% psychological distress<br>- 6.9% PTSD symptoms
Bowen et al. (2014) [53]	South Africa	General construction	n = 676	Likert Scale	Stress	33% high stress
Bowers et al. (2018) [30]	Australia	General construction	n = 1124	Kessler 10	Psychological distress	32.5% moderate psychological distress (K10 16-21)<br>21.4% high psychological distress (K10 22-29)<br>6.3% severe psychological distress (K10 30-50)<br>(Moderate to Severe levels 2x Australian population)
Chapman et al. (2020) [54]	Australia	General construction	n = 486	Kessler 10	Psychological distress	22.9% moderate psychological distress (K10 score 16–21)<br>16.1% psychological distress (K10 score 22+)
Dennerlein et al. (2021) [28]	USA	General construction	n = 259	Kessler 6	Psychological distress	32% moderate psychological distress (K6 score 5-12)<br>7% severe psychological distress (K6 score ≥ 13)
Dong et a. (2015) [32]	USA	General construction	n = 1114	ICD-9 diagnosis codes	Depression disorders	11% depression disorder
Dong et al. (2022)[55]	USA	General construction (male ≥18 years of age)	n = 12034	Kessler 6 Patient Health Questionnaire , PHQ 9	Psychological distress<br>Suicide ideation	23.8% moderate psychological distress (K6 score 5–12)<br>5.8% severe psychological distress (K6 score ≥13)<br>2.5% suicide ideation
Huang et al. (2020) [36]	China	General construction	n = 18300	CES-D	Depression symptoms	30% depression symptoms

		(manual labourers)				
Jacobsen et al. (2013) [6]	USA	General construction	n = 172	Hopkins Symptoms Checklist, HSCL-25<br>Kessler 6	Mental distress<br>Psychological distress	15.7% Mental distress (HSCL-25 score ≥ 1.50)<br>4.7% severe psychological distress (K6 score ≥13)
Kamardeen & Sunindijo (2017) [35]	Australia	General construction	n = 289	Depression, Anxiety and Stress Scales, DASS-21	Anxiety symptoms<br>Depression symptoms<br>Stress	36.4 % anxiety symptoms<br>32.5% depression symptoms<br>28.6% high stress
Lim, S et al (2017) [7]	Korea	General construction	n = 430	STAI-T<br>CES-D	Anxiety symptoms<br>Depression symptoms	41.6% anxiety symptoms<br>37.6% depression symptoms
Palaniappan et al. (2022) [34]	Singapore	General construction	n = 348	Depression, Anxiety and Stress Scales, DASS-21	Anxiety symptoms<br>Depression symptoms<br>Stress	29% depression symptoms<br>37% anxiety symptoms<br>33% high stress
Ross et al. (2022) [38]	Australia	General construction (apprentice)	n = 1401	Patient Health Questionnaire, PHQ 9	Suicide ideation	29.4% suicide ideation
Sellenger & Oosthuizen (2017) [56]	Australia	General construction (remote workers)	n = 113	Kessler 10	Psychological distress	22.9% moderate psychological distress (K10 16-21)<br>17.1% high psychological distress (K10 22-29)<br>8.6% severe psychological distress (K10 30-50)
Tyler et al. (2022) [37]	Australia	General construction	n = 1721	Patient Health Questionnaire, PHQ 9	Suicide ideation	7.3% suicide ideation
Wang, C et al. (2017) [57]	Malaysia	General construction	n = 201	Medical questionnaire	Anxiety symptoms<br>Depression symptoms	68% anxiety symptoms<br>72% depression symptoms
Wulsin, L et al. (2014) [31]	USA	General construction:<br>1. General contractors<br>2. Heavy civil contractors<br>3. Specialty subcontractors	1. n = 1978<br>2. n = 1043<br>3. n = 4408	ICD-9 diagnosis codes	Depression disorders	1. 9.7% depression disorder (GC)<br>2. 7.5% depression disorder (HCC)<br>3. 11.7% depression disorder (SSC)
Zhang et al. (2023) [9]	China	General construction	n = 336	Depression, Anxiety and Stress Scales, DASS-21	Anxiety symptoms<br>Depression	33.6% anxiety symptoms<br>36.6% depression symptoms<br>14.6% high stress

	symptoms
	Stress
